# Predictors of serological failure after treatment in HIV-infected patients with early syphilis in the emerging Era of universal antiretroviral therapy use

**DOI:** 10.1186/1471-2334-13-605

**Published:** 2013-12-26

**Authors:** Sadao Jinno, Bryan Anker, Parveen Kaur, Claire C Bristow, Jeffrey D Klausner

**Affiliations:** 1 Department of Medicine, AIDS Healthcare Foundation, Los Angeles, CA USA; 2 Fielding School of Public Health, University of California, Los Angeles, CA USA; 3 Division of Infectious Diseases, Program in Global Health, University of California, Los Angeles, CA USA; 4 1300 N. Vermont Ave. #407, Los Angeles, CA 90027, USA

**Keywords:** Syphilis, HIV, Serological failure, Benzathine penicillin

## Abstract

**Background:**

The optimal treatment of early syphilis (primary, secondary and early latent) in HIV-infected patients remains controversial. The Center for Diseases Control STD Treatment Guidelines recommended 1 dose of benzathine penicillin G (BPG) regardless of HIV infection. However, many providers modify the treatment for early syphilis.

**Methods:**

We performed a retrospective chart review of all cases of early syphilis with positive serologic test results in HIV-infected patients from May 2006 to May 2011 in 2 large, urban HIV clinics. Early syphilis includes primary, secondary, and early latent syphilis. Serological failure was defined as a lack of 4-fold decrease in rapid plasma reagent (RPR) titers 9 to 12 months after syphilis treatment. Patients whose RPR titers decreased after treatment and subsequently increased 4-fold at 9 to 12 months were excluded from the analysis of serological response because of possibility as “reinfection”. Baseline characteristics were tested as predictive factors of serological failure using a univariate and multivariate logistic regression model, respectively.

**Results:**

Of 560 patients with confirmed cases of early syphilis, 51 (9.0%) experienced serological failure. Multivariate logistic regression modeling demonstrated that the predictive factors associated with serological failure after early syphilis treatment were baseline RPR titer ≤ 1:16 (OR 3.91 [95% CI, 2.04-7.47]), a previous history of syphilis (OR 3.12 [95% CI, 1.55-6.26]), and a CD4 T-cell count below 350 cells/ml (OR 2.41 [95% CI, 1.27-4.56]). Of note, type of syphilis treatment (1 dose versus 3 doses of BPG) did not appear to affect the proportion of serological failure (4% versus 10%, P = 0.29), however the power of this study to detect small differences was limited.

**Conclusions:**

HIV-infected patients with baseline RPR titer ≤1:16, syphilis history, and/or a CD4 T-cell count <350 cells/ml should be closely monitored for serologic failure after early syphilis treatment. This study did not detect a substantial difference between treatment with > 1 dose of BPG and decreased frequency of serological failure, supporting the current recommendation that one dose of BPG is adequate treatment for early syphilis in HIV-infected patients.

## Background

Despite a significant decrease in syphilis cases in the United States during the 1990s, syphilis incidence has risen dramatically since 2000 [1]. Its prevalence remains especially high in HIV-infected men who have sex with men (MSM). Previous studies demonstrated that HIV-infected patients with syphilis may be more likely to experience serological failure as compared to non-HIV-infected patients [2,3]. A recent survey reveals that infectious disease specialists often treat secondary syphilis among HIV-infected patients with 3 doses of benzathine penicillin G (BPG) rather than 1 dose of BPG (62% vs 32%) even though the 2010 Centers for Disease Control and Prevention (CDC) STD Treatment Guidelines recommended treatment for early syphilis remains a single 2.4 million units intramuscular dose of BPG in both HIV-infected and HIV-uninfected patients [4,5]. The practice of 3 doses of BPG injection for HIV-infected patients with early syphilis may be motivated by the concern that 1 dose of BPG treatment might result in worse serological and/or clinical complications including central nervous system involvement. However, these approaches have theoretical benefit only, with no evidence of significant clinical benefit vis-à-vis the added cost.

Several studies have demonstrated that certain risk factors are predictive of an increased risk for serological failure [6-8]. For example, Ghanaem et al. found that a CD4 cell count of <200 cells/ml and/or the lack of antiretroviral therapy (ART) was associated with an increased risk of serological failure rates among HIV-infected patients with syphilis [6]. Others have demonstrated that baseline rapid plasma reagin (RPR) titers, syphilis stage, age, and number of sex partners may be associated with an increased risk of serological failure in HIV-negative patients with early syphilis [7,8]. However, minimal information is available for other risk factors such as treatment regimens, especially a total dose of BPG, or the degree of immnosuppresion in the emerging era of universal ART use. Therefore, we hypothesized that treatment regimens and immunosuppression might contribute to serological response in HIV-infected patients who are on ART. The objective of this study was to assess clinical characteristics and treatment regimens associated with serological response among HIV-infected patients with early (primary, secondary and early latent) syphilis.

## Methods

### Setting

AIDS Healthcare Foundation is one of the largest community-based HIV/AIDS medical providers in the nation, serving over 5000 patients in Los Angeles, California, USA. We performed a retrospective review of medical records to identify all cases of early syphilis with positive serologic test results in HIV-infected patients from May 2006 to May 2011 in our 2 large urban clinics in Los Angeles. The AIDS Healthcare Foundation human research protection approved the project. During the study period, RPR testing was performed on all patients every 3 months. Inclusion criteria are as follows: (1) serologic follow-up performed 9 to 12 months after syphilis treatment. (2) RPR titers are ≥ 1:4 at the time of diagnosis. (3) Clear documentation of the patient’s early syphilis stage as primary, secondary or early latent syphilis and type of syphilis therapy received within 2 weeks of diagnosis. (4) In patients with syphilis history, a more than 4 fold increase of RPR titer was required to diagnose a new infection. (5) Positive RPR results were confirmed using fluorescence treponemal antibody absorption test. Patients were excluded from the analysis for the following reasons: no follow-up testing within the period, no documentation of treatment, patients with diagnosis of late latent syphilis or syphilis of unknown duration, cases of primary syphilis with nonreactive serologic characteristics at the time of treatment. For those who satisfied inclusion criteria, medical record review was further performed to obtain information about patient characteristics, medical conditions, age, race, gender, syphilis stage (primary, secondary, early latent), baseline RPR titer, CD4 T-cell count, plasma HIV viral load, ART use, syphilis history, current amphetamine use, syphilis treatment type such as 1 or more doses of BPG, and history of other sexually transmitted diseases. CD4 T-cell count and plasma HIV viral load were analyzed at the time of syphilis diagnosis. CD4 T-cell count, HIV viral load, and age were evaluated as a dichotomous variable (CD4 T-cell count <350 cells/ml or ≥350 cells/ml, detectable HIV viral load as ≥400 copies/ml or undetectable <400 copies/ml, age ≥40 or <40). ART use was defined as the use of at least 3 antiretroviral drugs, from 2 different classes for a duration of > 6 months.

Disease stage was classified on the bases of patient history and clinical examination. Patients were considered to have primary syphilis (ulcer at anogenital or oropharyngeal sites and positive serology), secondary syphilis (mucocutaneous and/or skin lesions typical for syphilis and positive serology), or early latent syphilis (no clinical sings of syphilis and newly positive serology within the past year or ≥ 4-fold increase in RPR titer within the past year).

### Study outcomes/definition of serological failure

The primary outcome was response to syphilis treatment, determined on the basis of change in RPR titers after treatment. Serological failure was defined as a lack of a 4-fold decrease in RPR titers at 9 to 12 months after initial treatment or a titer pattern consistent with re-infection. Because we wanted to include only patients likely to have had true treatment failure, patients whose RPR titers decreased after treatment and subsequently increased 4-fold at 9 to 12 months were excluded from the analysis of serological response as “reinfection”.

### Data analysis

To identify potential predictors of developing serologic failure, we compared demographic and clinical characteristics of patients with serological failure to those with serological cure using univariate logistic regression. Demographic and clinical characteristics for serological failure were selected a priori based on previous literature, clinical plausibility, and ease of clinical collection. Combination of risk factors significant in univariate analyses were assessed and were selected in a stepwise algorithm, with variables entered and excluded for P > .025. Post-hoc power analysis tests were performed, with an alpha of .05, in order to assess the minimum level of effect that would be detected. Data were analyzed using SAS, version 9.3 (SAS Institute).

## Results

During the study period, a total of 777 HIV-infected patients with early syphilis were identified. Among the 777 patients, 217 were excluded due to: lack of data (84 patients), loss of follow-up (48 patients), incomplete chart (37 patients), reinfection (28 patients), or because RPR titer were 1:4 (20 patients). “Reinfection” was defined as a RPR pattern of decreased RPR titers after treatment and subsequently 4-fold increase between 9 to 12 months.

Table 1 shows the characteristics of the 560 patients included in the analysis. Of 560 persons with confirmed cases of early syphilis, 486 (87%) were on ART; 96% of patients were MSM; 51% had syphilis history; 32% uses methamphetamine actively; 21% had hepatitis B or C; 72% achieved undetectable viral load as defined below 400 copies/ml. The median CD4 T-cell count was 491. Most (60%) presented with early latent syphilis. The median RPR titer was 1:64. Majority of patients (78%) received 3 doses of BPG injection. A total of 51 patients (9.0%) experienced serological failure.

**Table 1:** Clinical characteristics of 560 HIV-infected patients with early syphilis

**Characteristic**	**No. (%)**
Age, median year [IQR]	40 [34,45]
Male sex	556 (99)
Race	
Hispanic	249 (44)
White	214 (38)
Black	62 (11)
Other/Unknown	35 (6)
MSM	541 (96)
Methamphetamine use	191 (34)
Hepatitis B or C	115 (21)
Syphilis history	287 (51)
Other STD^a^	419 (75)
Median RPR titers [IQR]	1:64 [1:32,1:128]
<1:32	134 (24)
≥1:32	426 (76)
ART	486 (87)
CD4 T-cell count	
Median CD4 T-cell count, cells/ml [IQR]	491 [345,683]
<350 cells/mL	144 (26)
≥350 cells/mL	415 (74)
HIV RNA	
Median HIV RNA level, copies/ml [IQR]	ND [ND,631]
Not detectable^b^	400 (72)
Detectable	160 (28)
Early syphilis stage	
Primary	77 (14)
Secondary	146 (26)
Early latent	337 (60)
Treatment regimen	
3 doses of benzathine penicillin	434 (78)
1 dose of benzathine penicillin	47 (8.4)
Doxycycline	78 (14)
Azithromycin	1 (0.2)

Data are number (%) of patients, unless otherwise indicated. IQR, interquartile range; MSM, men who have sex with men; RPR, rapid plasma regain test; STD, sexually transmitted disease; HIV, human immunodeficiency virus; DM, diabetes mellitus; ART, combination antiretroviral therapy; ND, not detectable.

^a^Other STD include documented Neisseria gonorrhoeae, chlamydia trachomatis, herpes, and genital warts.

^b^Defined as <400 copies/mL.

Table 2 shows the characteristics of the 560 patients included in the univariate analysis. By univariate logistic regression analysis, multiple factors were associated with serological failure, including race (black), syphilis history, RPR titers ≤ 1:16, and a CD4 T-cell count <350 cells/ml. There was a risk difference of -5.6% (P = 0.29, 95% CI: -12.1, 0.8) between the risk of serological failure between the two groups of BPG for syphilis treatment (1 dose versus 3 doses of BPG). However, post hoc power analysis identified based on our observed allocation of BPG treatment (47 patients received 1 dose of BPG; 434 patients received 3 doses of BPG), demonstrated that a statistically significant difference would only be observed with a risk difference of about 13.5% or greater. Syphilis stage, methamphetamine use, age, ART use, and HIV viral load were not significantly associated with serological failure. Neither hepatitis B or C infection was associated with serological failure (odds ratio [OR] 1.07; 95% confidence interval [CI], .53- 2.16).

**Table 2:** Clinical characteristics and predictors of serological failure among 560 HIV-infected patients with early syphilis

**Characteristic**	**Patients with serological failure (*n*** **= 51)**	**Patients with serological cure (*n*** **= 509)**	**Odds ratio (95% CI)**
Age, years			
<40	24 (8.8)	250 (91.2)	0.89 (.46-1.79)
≥40	27 (9.4)	259 (90.6)	1
Race			
Hispanic	22 (8.8)	227 (91.2)	1
White	17 (7.9)	197 (92.1)	0.89 (.46-1.73)
Black	11 (17.7)	51 (82.3)	2.23 (1.02-4.88)
Other/Unknown	1 (2.9)	34 (97.1)	0.30 (.04-2.33)
Methamphetamine use	19 (9.9)	172 (90.1)	1.16 (.64-2.11)
Hepatitis B or C	11 (9.6)	104 (90.4)	1.07 (.53-.16)
Syphilis history	40 (13.8)	249 (86.2)	3.80 (1.91-7.56)
Other			
STD^a^	39 (9.3)	380 (90.7)	1.10 (.56- 2.17)
RPR titer			
<1:32	23 (17.0)	112 (83.0)	2.91 (1.61-5.25)
≥1:32	28 (6.6)	397 (93.4)	1
ART	44 (9.1)	442 (90.9)	0.95 (.41- 2.20)
CD4 T-cell count			
<350 cells/mL	23 (16.0)	121 (84.0)	2.63 (1.46-4.74)
≥350 cells/mL	28 (6.7)	388 (93.3)	1
HIV RNA			
Undetectable^b^	33 (8.2)	369 (91.8)	1
Detectable	18 (11.4)	140 (88.6)	1.44 (.78-2.64)
Early syphilis stage			
Primary	9 (11.7)	68 (88.3)	1
Secondary	10 (6.8)	136 (93.2)	0.56 (.22-1.43)
Early latent	32 (9.5)	305 (90.5)	0.79 (.36-1.74)
Treatment regimen			
3 doses of benzathine penicillin	43 (9.9)	391 (90.1)	1
1 dose of benzathine penicillin	2 (4.3)	45 (95.7)	0.40 (.10-1.73)
Doxycycline	6 (7.7)	72 (92.3)	0.76 (.31-1.85)
Azithromycin	0 (0)	1 (100)	NA

Data are no. (%) patients. OR, Odds Ratio; CI, confidence interval; RPR, rapid plasma regain test; STD, sexually transmitted disease; HIV, human immunodeficiency virus; DM, diabetes mellitus; ART, antiretroviral therapy.

^a^Other STD include documented Neisseria gonorrhoeae, Chlamydia trachomatis, herpes, and genital warts.

^b^Defined as <400 copies/mL.

Using a *P* value cutoff of .025, a model identifying serological failure was generated. This multivariate logistic regression modeling demonstrated that the predictive factors associated with serological failure after early syphilis treatment were a CD4 T-cell count below 350 cells/ml (OR 2.41; 95% [95% CI, 1.27-4.56]), syphilis history (OR 3.12 [95% CI, 1.55-6.26]), and baseline RPR titer ≤ 1:16 (OR 3.91 [95% CI, 2.04-7.47]), independent of other characteristics.

## Discussion

Our study was the large systematic evaluation assessing type of syphilis treatment associated with serological response in HIV-infected patients with early syphilis. Our population had a very high risk of past and current syphilis exposure as half had a history of syphilis infection. We found that overall rate of serological failure after therapy was low (9%) in our HIV-infected patients, and the duration of syphilis treatment (1 dose versus 3 doses of BPG) did not affect the proportion of serological failure (4% in a group of 1 dose of BPG versus 10% in a group of 3 doses of BPG) while our small number of serologic failure limited the power to detect a small difference in serological failure rates between individual who received one dose and three doses of BPG. However, based on our sample size and treatment distribution, we can exclude the difference greater than 13.5%. Our results have important clinical implications therefore it is important to assess whether there is a true lack of effect. HIV-infected patients with early syphilis often receive 3 doses of BPG. However, BPG may have adverse effects including allergic reaction, neurotoxicity, neutropenia, and local pain [9-13]. Therefore, keeping the dose of 2.4 million units of BPG as per the CDC guidelines may be beneficial.

Limited data is available regarding a total dose of BPG for early syphilis in HIV-infected patients, possibly causing substantial variation in the management of early syphilis for HIV-infected patients among clinicians [4]. In pre-ART era, a randomized trial compared 2.4 MU of BPG to enhanced therapy with a 10-day course amoxicillin and probenecid for early syphilis among HIV-infected and non HIV-infected patients [14]. The rates of serological failure did not differ according to treatment group, but it is not entirely clear whether the rate of serological failure differed in HIV-infected patients since most of participants (81%) were not HIV-infected. More recently, a retrospective study from England, involving 77 HIV-infected patients with early syphilis, described that 3 doses of BPG does not significantly alter the serological cure rate as compared to 1 dose. However, the study was underpowered with a result of a wide confidence interval (78.9% (95% CI 68.0 – 89.8) versus 64.1% (95% CI 45.0 – 73.2), *P* > 0.05), and did not exclude patients who had been lost to follow-up and/or re-infected. Furthermore, the majority of previous studies suggested that HIV status is not associated with serological response to therapy [3,7,15-19]. For example, a large scale cohort study conducted in Zambia and Rwanda, involving 933 HIV-infected and 388 HIV-uninfected patients, demonstrated that HIV infection did not impact the likelihood of serologic response to therapy in a multivariate analysis (OR, 1.001; *P* = .995) [15]. Their median CD4 T-cell count was 367 cells/ml in Zambia and 446 cells/ml in Rwanda. Although there is no randomized controlled trial comparing therapy of 1 dose with >1 doses of BPG in HIV-infected patients with early syphilis, our findings and the review of existing evidence support that one dose of BPG is adequate treatment.

Of interest is our finding that persons with CD4 T-cell count <350 cells/ml were more likely to develop serological failure, independent of type of treatment (OR 2.41 [95% CI, 1.27-4.56]). This finding is consistent with previous studies that demonstrated HIV-co-infected patients with low CD4 T-cell count showed significantly slower treatment response [6,7]. We speculate that HIV-infected patients with more immunosuppression may respond to treatment slowly rather than experiencing treatment failure. In fact, among the 23 patients with CD4 T-cell count <350 cells/ml who developed serological failure, 20 (87%) achieved serological cure 12–24 months after treatment, and we did not observe central nervous involvement in 15 out of the 20 patients after evaluation of cerebral fluid examination (5 patients refused cerebrospinal fluid examination).

One of the strongest predictors of developing serological failure in our cohort was RPR titer ≤1:16 (OR 3.91 [95% CI, 2.04-7.47]). This finding agrees with the observation that patients with low titers may be at risk of serological failure after treatment as alluded by Sena et al. in HIV negative populations [8]; whereby the authors postulated that high VDRL titers may reflect a more robust immunological response associated with better clearance of the organism. Additionally patients with lower titers may have a longer duration of infection and the response to treatment may be delayed as metabolic activity of syphilis could be lower in later stages of infection [20,21]. Or 4-fold decline in titer may simply be easy to occur in patients with higher titers.

Our study has several limitations. First, a selection bias is possible because 28% of patients were excluded due to lack of data or loss of follow-up. Secondly, the number of patients treated with one dose of BPG was low (n = 47, 8.4%), and thus a lack of statistical power cannot be excluded as the cause of the observed lack of effect of dosage on serological failure. However, the study did have adequate power to detect the risk difference of 13.5% or greater. Third, our study was based on medical record review and document may have been incomplete. Fourth, we excluded patients with primary syphilis whose serology was negative as well as those whose titer were <1:4. Therefore, our results may not be generalizable to those who have primary syphilis with negative serology or whose titers are <1:4. Finally, unmeasured potential confounding variables must be considered as limitations of all retrospective cohort studies such as inadvertent exposure to other efficacious antibiotics against syphilis.

## Conclusion

In conclusion, HIV-infected patients with baseline RPR titer ≤ 1:16, syphilis history, and/or a CD4 T-cell count <350 cells/ml should be closely monitored for serological failure after early syphilis treatment. Treatment with > 1 dose of BPG was not associated with a large difference in frequency of serological failure. Further research must be conducted to evaluate the current recommendation that one dose of BPG is adequate treatment for early syphilis in HIV-infected patients.

## Competing interests

They authors declare that they have no competing interests.

## Authors’ contributions

SJ collected the clinical information of cohort data and participated in the design of the study, performed statistical analysis, and prepared manuscript. BA collected the clinical information of cohort data. PK prepared manuscript. CCB performed statistical analysis and prepared manuscript. JDK conceived of the study, and participated in its design and coordination and helped to draft the manuscript. All authors read and approved the final manuscript.

## Pre-publication history

The pre-publication history for this paper can be accessed here:

http://www.biomedcentral.com/1471-2334/13/605/prepub
